# The Hospital Dietary

**Published:** 1916-12-02

**Authors:** Elliott P. Joslin

**Affiliations:** Associate Professor of Medicine, Harvard University, Boston.


### THE HOSPITAL DIETARY.*

### A Too Much Neglected Study.

By Dr. ELLIOTT P. JOSLIN, Associate Professor of Medicine, Harvard University, Boston.

As I read your programme and recognised the names of well-known specialists in hospital management, it was with much diffidence that I ventured to record any opinions of a member of the staff about the hospital dietary. This feeling gained force while my paper took form, because most of the points which I planned to make were criticisms of hospital diets. But when I came to consider closely the dietary of several of our Boston hospitals, to my gratification, though also somewhat to my embarrassment, I found that most of the suggestions contemplated were already in use, and the criticisms about to be proposed were no longer applicable. However, it may do you no harm, you who usually receive complaints of the hospital dietary, to hear various qualities in it held up to commendation.

The food in the hospital dietary should be abundant, plain, simply prepared, inexpensive, not susceptible to waste, and of such a character that it can be easily digested. These attributes are axiomatic, but I expect that your Committee on Hospital Standardisation will not find it easy to settle the caloric value of the diet, though the data which they accumulate will be most valuable. Of course, the experience in other institutions than hospitals will be very helpful, and I anticipate that the present European crisis will furnish data far more useful than any hitherto obtained. Simplicity in a hospital diet is important because false ideas as to the character of the food necessary for health should not be encouraged in patients. Neither should methods of cooking be employed which are impossible of imitation in small dwellings. The necessity for an inexpensive diet and one not susceptible to waste need not be emphasised before this body, but it will be appropriate to combat a common impression that food should be excessively simple or really of a nature of soft solids to be digestible. In reality, the digestion of most people is admirable, and any suitably cooked food is well borne. One of the best proofs of this is the absence of indigestion among diabetic patients under the former plan of forced feeding or the present low diet. All these qualifications of the general hospital dietary I found existed at one of the Boston hospitals, and undoubtedly are more universal than I supposed.

Especially to be commended was the variety of the food offered the patients, possibly an inheritance of Dr. Howard's plan of rotating the standard portions of the diet in some other number of days than seven.

Stews and boiled meats rightly figured prominently in the menus. Meats so prepared are to be commended because they are soft and do not require good teeth. Furthermore, stews and boiled meats can be kept hot and moist far more easily than roasts, can be served with vegetables and simple condiments, and thus made especially appetising. Stews abolish the need of soups. Incidentally, such methods lessen waste in the cooking and serving. I deprecate the use of thin broths; they could be quite as attractive and of some nutritive value if thickened. They set a bad example to the patient.

The opportunity of the coast cities to utilise fish freely is appreciated. Since meat, fish, and eggs are not given the patient at night, there remain but fourteen other meals a week, and of these fourteen meals I noted that at least four contained fish as a basis. It is not to be wondered at that protein in a leguminous form figured quite as frequently in the diet as the Boston public would be expected to demand.

The early light hospital suppers do more to give the cardiac patients good nights than they or their physicians suspect. The increased use of fruit now in the hospital diet as contrasted with former times reflects the general tendency of the community, and is especially appropriate. The discrimination shown by the hospital authorities in the selection of vegetables was gratifying. Thus, at no time in the course of several weeks did I detect that more than one white vegetable was served at a meal. Potato, rice, or macaroni was invariably combined with one of the courses, or green vegetables. Cabbage might be more freely employed, especially in surgical cases. Of course, the whole success of its use would lie in the way it was prepared. Why not use cabbage instead of bran and cathartics? It would seem as if bananas might be used more. There is no more waste to a banana than to an egg. The caloric value is high. If suitably prepared, the banana is easily digested. It affords variety. It has a moral value. Did you ever know an individual to commence a banana that he did not finish it? It is an easy way to introduce sugar and milk or cream into the diet. To the uninitiated, ripe olives do not appear expensive if purchased in bulk.

The hospital diet is designed to cure the patient. Even more than rest, it constitutes the chief therapeutic agent.

### SPECIAL DIETS.

These must be the bane of the hospital dietician's life. Each succeeding group of physicians devises a new "special diet." Such originality should be discouraged. Often these diets differ very slightly from one another, and much labour might be spared if yearly they were revised by common agreements of the medical and surgical staffs. The dates of the revisions should be printed upon the diet slips. Perhaps these special diets do not deserve as much attention, for as a matter of fact the day is coming, even if it has not arrived, in which we shall see the passing of the standard special diet. Its place is being taken by the universal adoption of individualistic prescribing of food.

* A paper read at the recent Conference of the American Hospital Association.

Already we hear less and less of the nephritic diet, the diabetic diet, the typhoid diet, the obesity diet, the low-protein and low-salt diet, and the gastric-ulcer diet, but more and more of how many calories, how much protein, carbohydrate, fat, the patient should receive. There is no such thing as a diabetic diet, and no such thing as an obesity diet. We speak of a hyperacidity diet, but no part of the hospital diet should be such that it would cause hyperacidity. The size, age, and special requirements of each patient must be considered, as well as whether he is abed or up and dressed. Such a plan involves extra help, more dieticians, better training of nurses, but the education of the community is progressing. Almost anyone now, even if he has not a mechanical bent, can run an automobile and almost any modern nurse ought to be able to put mental as well as physical energy into the selection and serving of her patient's dinner. The extra help required in arranging the diets will be more than offset by the saving of food and the shortening of the stay of the patient in the hospital.

### Complaints with the Hospital Dietary.

Fault is frequently found with the diet. I doubt if there is any exaggeration in the statement that if such complaints were sifted it would be found that 90 per cent. related to the methods of serving the food after it left the kitchen. If Dr. Howard's dictum of "Let the 'hots' be hot and the 'colds' be cold" were followed, most complaints would vanish. Too much pains cannot be taken in the serving of the food. Even hot, deliciously cooked roast beef provokes no favourable response if served dry. The fact that it is not the quantity or the quality of the food which provokes criticism, but rather the method in which it is served, should ever be borne in mind. In private hospitals this is still more important. Sometimes it seems as if the nurses had received no instruction in serving the food. Private patients resent this bitterly, for the waitresses in their homes are often quite neat and deft in their methods. Do you, as hospital superintendents, realise as you live sumptuously at Bellevue-Stratfords, wherever you go, that some of your nurses may not have been so fortunate or not have been accustomed to such dainty methods of service?

### Cost and Waste.

The problem of cost and waste may be divided into two branches—that connected with the food before it has reached the kitchen, and that connected with its preparation and until it has left the hospital. It would be unwise for me to attempt here to express any opinion about the former, but as to the latter I feel less reticence. The returned tray of a diabetic patient is a model in dietetic economy. If any of you have looked at the trays returned from diabetic patients, you will see that the problem of waste is eliminated, for these patients eat everything given them. Indeed, they will eat all the salt in the salt-cellar if allowed to do so. If there is no waste in the diabetic diet after it reaches the patient, why should there be in any other diet? Of course it must be granted that these patients, under modern conditions, are often underfed, but I do not believe that this by any means accounts for the whole story. The more important fact is that they are served with great care, and no more food given them than is required. In other words, dietetic individualism is carried to the extreme. This involves increased labour on the part of someone, but I believe that the amount of food saved would more than compensate for the extra wages expended. With the increased cost of food this individualistic feeding will come soon. The fact that the custom is growing of serving individual portions of food to the help shows this tendency. So far as waste in a diet is concerned, however, there is little question but that the experiences now being acquired in the European war will furnish us much of value, and lessons of this character will mitigate the many losses which now seem irreparable.

A second measure which will reduce the cost of the hospital dietary and cost per patient is the suitable grouping of similar cases. I cannot conceive how a hospital can persist in having typhoid patients scattered throughout all the medical wards, when these might be grouped together in a few rooms. Nor can I understand how diabetic patients can be allowed to remain under the care of many nurses, who may have only one case a year to treat, when these might be grouped under the ready supervision of a single nurse. I have had a wide opportunity of observing the two methods, both in private and in public hospitals. The easy way in which the diabetic dietetic problem is handled in a private hospital, where the responsibility for the diet rests upon one nurse with nurses serving under her for periods of three months, compared with the management of a much smaller number of diabetic patients in another hospital, where frequently the nurses have never had a diabetic case under their care, daily forces this upon me.

Finally, the prevention of waste should be stimulated in patients. Patients should be taught economic habits in dealing with food. They should not be allowed to see food wasted. Too much praise cannot be given to nurses who are careful to conserve the food supply. The active interest of the staff should be frequently invoked.

The third means of reducing the cost of the food supply you are already seeking by your Committee on Hospital Standardisation. I wish I could hear the report of that committee. Surely it will be most edifying. But it should be applicable in greatest measure to the smaller hospitals. The large hospitals—hospital towns, almost cities, you might call them—existing in the large cities can afford experts on hospital efficiency, but the little hospitals cannot, and your committee should do this work for them. Just as soon as the smaller hospitals have put before them the cost of food per patient, then they will be in a position to judge whether they are properly conserving their resources. The Government has standardised railroad accounts, and your committee should do the same for hospitals.

(*To be continued.*)

